# Impact of the use of an endorectal coil for 3 T prostate MRI on image quality and cancer detection rate

**DOI:** 10.1038/srep40640

**Published:** 2017-02-01

**Authors:** Josephin Gawlitza, Martin Reiss-Zimmermann, Gregor Thörmer, Alexander Schaudinn, Nicolas Linder, Nikita Garnov, Lars-Christian Horn, Do Hoang Minh, Roman Ganzer, Jens-Uwe Stolzenburg, Thomas Kahn, Michael Moche, Harald Busse

**Affiliations:** 1 Department of Diagnostic and Interventional Radiology, Leipzig University Hospital, Liebigstraße 20 Leipzig, Germany; 2 Institute of Pathology, Leipzig University Hospital, Liebigstraße 24 Leipzig, Germany; 3 Department of Urology, Liebigstraße 20 Leipzig University Hospital, Leipzig, Germany

## Abstract

This work aims to assess the impact of an additional endorectal coil on image quality and cancer detection rate within the same patients. At a single academic medical center, this transversal study included 41 men who underwent T2- and diffusion-weighted imaging at 3 T using surface coils only or in combination with an endorectal coil in the same session. Two blinded readers (A and B) randomly evaluated all image data in separate sessions. Image quality with respect to localization and staging was rated on a five-point scale. Lesions were classified according to their prostate imaging reporting and data system (PIRADS) score version 1. Standard of reference was provided by whole-mount step-section analysis. Mean image quality scores averaged over all localization-related items were significantly higher with additional endorectal coil for both readers (p < 0.001), corresponding staging-related items were only higher for reader B (p < 0.001). With an endorectal coil, the rate of correctly detecting cancer per patient was significantly higher for reader B (p < 0.001) but not for reader A (p = 0.219). The numbers of histologically confirmed tumor lesions were rather similar for both settings. The subjectively rated 3-T image quality was improved with an endorectal coil. In terms of diagnostic performance, the use of an additional endorectal coil was not superior.

Multiparametric magnetic resonance imaging (MRI) of the prostate has become a reliable technique for the detection, localization and characterization of prostate cancer (PCa). The combination of morphologic and functional MRI information is largely accepted as the best imaging correlate for PCa diagnostics^1^. The diagnostic potential of such an imaging approach has already been demonstrated by different clinical research groups^2^,^3^. Despite the wide use of multiparametric MRI, there is still no consensus about the minimal and optimal imaging requirements.

The European Society of Urogenital Radiology has therefore issued guidelines that describe proper multiparametric MRI protocols and clinical indications^4^. There is also an ongoing discussion about the minimum equipment required for proper MRI diagnostics, in particular with increased clinical use of 3-T MRI systems. While many studies at 1.5 T have shown a clear diagnostic benefit with an additional endorectal coil (ERC)^5^,^6^,^7^, the need for an ERC at 3 T is still discussed controversially raising the question whether the extra efforts and discomfort for the patient are justified^8^,^9^,^10^.

Therefore, the purpose of this study was to compare image quality with respect to both localization and staging-related items, as well as PCa detection rate and localization between surface coils (SC) only and in combination with an ERC at 3 T within the same patients.

## Methods

### Patient Characteristics

Data collection, analysis and publication were approved by the Institutional Review Board (IRB) of the Leipzig University Faculty of Medicine, Leipzig, Germany (reference number 099-10-19042010-ff) and informed consent was obtained from all subjects. Methods were carried out in accordance with the approved guidelines.

This transversal analysis at a single academic medical center included 41 men who underwent 3-T T2- and diffusion-weighted imaging (T2WI, DWI) between October 2010 and September 2012 using SC only or in combination with an ERC prior to radical prostatectomy. General exclusion criteria were contraindications to either MRI (e.g., pacemaker or cerebral metal clips), or gadolinium-based MR contrast agents or ERC insertion (e.g., prior anorectal surgery, inflammatory bowel disease or high anal sphincter tension), as well as severe claustrophobia. MRI was performed 1–3 days (mean 1.1 days) before prostatectomy. The median time between the latest transrectal ultrasound-guided biopsy and MRI was 55 (range 11–119) days.

### MRI Protocol

MRI was performed in a 3-T system (Magnetom Trio, Siemens Healthcare, Erlangen, Germany) with the patient in supine position. The first part of the examination involved two surface coils only; a 6-channel body matrix coil placed ventrally at the pelvic level and selected elements of a 24-channel spine matrix coil integrated into the MR table. T2WI was based on fast spin-echo sequences (in-plane resolution IPR = 0.57 × 0.57 mm^2^, repetition and echo time TR/TE = 4,400–4,600/126 ms, slice thickness ST = 3.0 mm, slice gap SG = 0.6 mm, 19–22 slices, field of view FOV = 110 × 110 mm^2^, flip angle FA = 120–135°) and covered the whole prostate and seminal vesicles in both transverse and coronal planes. Transverse images were aligned perpendicular to the anterior rectal wall. DWI was performed with a single-shot echo planar imaging sequence (IPR = 1.0 × 1.0 mm^2^, TR/TE = 3000/85 ms, ST = 3.0 mm, SG = 0.6 mm, 19–22 slices, FOV = 250 × 250 mm^2^, FA = 90°) in transverse planes using b-values of 50, 500, 800 and 1,500 sec/mm^2^. Apparent diffusion coefficient (ADC) maps were calculated by using b-values 50, 500 and 800 sec/mm^2^. For lesion identification, readers also reviewed images acquired at a high b-value of 1,500 sec/mm^2^.

An ERC (eCoil, Medrad, Pittsburgh, USA) was added for the second part of the examination. It was placed in a lateral decubitus position after digital rectal examination. The ERC was filled with 30–40 mL of a perfluorocarbon compound (Perfluorooctyl bromide, ABCR GmbH, Karlsruhe, Germany) to minimize susceptibility artifacts. Peristalsis was routinely suppressed by intravenous administration of 1 mg of glucagon (Glucagen, Novo Nordisk, Gentofte, Denmark) given in two portions right before both examinations. This extended study protocol without and with ERC required between 40 and 50 minutes. The entire examination was tolerated well by all patients and no adverse events were reported.

### MRI Evaluation

Two readers had full access to all images on a standard radiological workstation (SIENET MV 1000, Siemens, Erlangen, Germany). They were blinded to the respective PSA levels, GS and histopathological classification of malignant tumors (TNM classification) but were aware that all subjects had biopsy-proven cancer. Reader A (M.R.-Z.), a radiologist with 6 years’ experience in general MRI diagnostics, had already read 70 endorectal prostate 3-T MRI cases while reader B (J.G.), a radiology resident with 2 years of general MRI experience, had read 160 such cases. For each patient, both MR images sets were anonymized and randomized into two equally sized groups. Images of the same patient were read in two separate sessions at least 6 weeks apart to reduce rater bias.

Image quality was evaluated by nine items following established criteria originally described by Heijmink *et al*.^10^: Five of them (a–e) focused on cancer localization and involved (a) discrimination between transitional and peripheral zone (TZ/PZ), (b) visibility of the PZ itself, (c) visibility of the TZ itself, (d) visibility of the lesion, (e) visualization of the internal architecture of the TZ. Four items (f–i) were related to tumor staging and included (f) delineation of the prostatic capsule, (g) visualization of the neurovascular bundle as well as (h) visualization of the rectoprostatic angle. For the last staging item, (i) presence of minimal capsular penetration, reader confidence was only assessed in the positive case. Reader confidence was generally rated on a five-point scale as very poor (1), poor (2), moderate (3), good (4), and very good (5).

The presence of image artifacts caused by motion or other sources (aliasing, susceptibility) was also evaluated on a five-point scale with image quality being severely affected (1), moderately affected (2), acceptable (3), hardly affected (4), or not affected (5). Lesions were classified according to PIRADS version 1. Both readers rated the presence of clinically significant disease as either highly unlikely (1), unlikely (2), unclear (3), likely (4), or highly likely (5) using PIRADS criteria for T2WI and DWI^4^. Circular regions of interest (ROI) were drawn within the boundaries of all cancer-suspicious lesions and the mean ADC was measured. Tumor localization was assigned by using a standardized 27-sector prostate MRI reporting scheme following the recommendations of the European Consensus Meeting in 2010^11^.

### Histopathological Work-up

All prostates were analyzed histologically immediately after surgical resection. Specimens were fixed in 10% neutral buffered formalin for approximately one week, the seminal vesicles were separated, and the prostate was sliced into 4 to 5-mm thick step sections perpendicular to the dorsorectal surface of the gland. After paraffin embedding and hematoxylin-eosin staining, all histological slices were evaluated by a single pathologist (L.-C.H., 14 years of experience in urogenital histology) who was blinded to MRI. Tumor foci were outlined on the microscopy slides. Primary and secondary lesions were tagged and characterized according to the Gleason grading system. All specimens were staged according to the TNM classification^12^. All slides were also digitized to simplify further analysis.

### Radiologic-Pathologic Correlation

After both readers had assigned their MRI scores, a supervisor correlated all MRI findings to the histopathological reference. This correlation is often difficult because of the mechanical (slicing) and chemical processing (fixation) of the specimens^13^. Radiologic-pathologic correlation was considered positive if the marked tumor focus on the whole-mount step section was within the same segment (position and side) and close (<5 mm) to the respective MRI focus. Morphological T2WI appearance of TZ, PZ and urethra as well as features like cysts and calcifications were used as landmarks. Only lesions with PIRADS scores ≥4 in either T2WI or DWI were considered to be suspicious for prostate cancer and included when histopathologically confirmed.

### Statistical Analysis

All statistical analyses were performed as two-sided tests (IBM SPSS 20, Chicago, IL). Statistical results were denoted with “ns” when not significant (p ≥ 0.05) or with a number of asterisks (*^,^ **^,^ ***) when significant (p < 0.05, p < 0.01 and p < 0.001, respectively). Whenever the selection of findings was specific to reader or setting, independent two-sample t-tests were used. This applied to the analysis of all PIRADS scores (different lesions) and staging item (i) presence of minimal capsular penetration (different choices). Differences between both settings for all other item-based ratings of image quality were analyzed by dependent t-tests with paired samples. Fisher’s exact tests (two-sided) were used to test for significant differences in the detection rates with respect to Gleason scores—low (5 or 6) vs. intermediate and high scores (7 or 8)—as well as localization—peripheral vs. transitional zone cancers. A McNemar test was used for the analysis of the rate of detecting prostate cancer in a patient.

## Results

Thirty-six of 41 patients showed organ-confined (stage pT2) disease. Five patients (12%) had locally advanced tumor (stage pT3), two of them with extracapsular extension (pT3a) and three with additional invasion of the seminal vesicles (pT3b). A detailed summary of patient characteristics and histopathological results is given in Table 1. Individual as well as mean image quality scores averaged over all localization-related items (a-e) were significantly higher (with ERC vs. SC only) for both readers (A: 4.0 vs. 3.4^***^, B: 4.5 vs. 3.4^***^). Corresponding individual and mean scores averaged over all staging-related items (f–h) were significantly lower for reader A (4.0 vs. 4.4^**^) and significantly higher for reader B (4.8 vs. 3.9^***^). Individual criterion (i) **presence of minimal capsular penetration** was not significant for reader A but for reader B who correctly detected all patients with histologically confirmed extracapsular tumor growth as well. Different types of artifacts were rated as hardly or not present by both readers independent of the specific setting. Table 2 summarizes the mean image quality scores assigned by both readers.

When using the additional ERC (vs. SC), cancer detection rates per patient were 88% for reader A (vs. 78%, p = 0.219) and 98% for reader B (vs. 68%^***^). The rates of correctly identified (histologically confirmed) individual tumor lesions were similar or higher for ERC acquisitions (slightly lower for reader A, 74% vs. 78%, and higher for reader B, 86% vs. 69%). Table 3 summarizes the detected PCa lesions with corresponding sizes and Gleason scores. Performance was not statistically different for low vs. intermediate plus high-grade cancers for both readers (p = 0.502 and 0.106, respectively). Tables 4 and 5 show the detection rates of PCa foci with respect to their localization (TZ vs. PZ) and their risk category (according to Thompson *et al*.^14^), respectively. Stratification against localization showed significant differences for reader B only, particularly in the peripheral zone (Table 4). Stratification against lesion size (diameter) was performed for reader A only, for which the remaining sample size was sufficiently large in both subgroups. For smaller lesions (diameter ≤ 10 mm), that reader identified 16/22 (true vs. suspected) cancer lesions (73%) with SC and 26/35 lesions (74%) with ERC. For larger lesions (diameter > 10 mm), the detection rates were 22/27 (81%) and 20/27 (74%).

In general, more lesions were rated as cancer-suspicious (PIRADS ≥ 4) when using an ERC (A: 61 vs. 48; B: 63 vs. 38). The mean PIRADS scores averaged over all correctly identified (histologically confirmed) PCa foci based on T2WI were not significantly different (higher) with an additional ERC (A: 4.6 vs. 4.4, p = 0.140; B: 4.4 vs. 4.2, p = 0.157). Using DWI, average PIRADS scores were also similar with both settings (A: 4.5 vs. 4.4, p = 0.396; B: 4.5 vs. 4.4, p = 0.577, ERC vs. SC). Mean ADC values across correctly identified tumor ROIs were higher with ERC (A: 800 vs. 711 mm^2^/sec, p = 0.125; B: 954 vs. 767 mm^2^/sec^**^).

**Figure 1:**
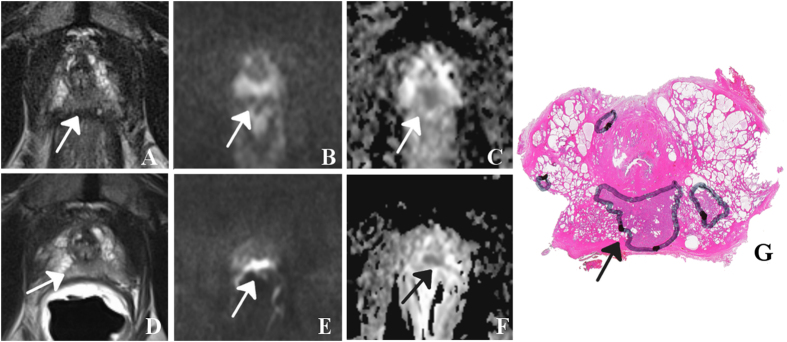
59 year-old patient with prostate-specific antigen level of 13 ng/ml and Gleason score 7 (3 + 4) prostate cancer (arrows) in the apex (segment 5p/11p): T2-weighted images (T2WI) with surface coils only (**A**) and with the addition of an endorectal coil (**D**) show a hypointense focus. The corresponding diffusion-weighted images (DWI, **B** and **E**, respectively) acquired with a b-value of 800 sec/mm^2^ reveal hyperintense focal areas with a corresponding (**C** und **F**, respectively) reduction on the ADC maps calculated from images with b-values of 50, 500 and 800 sec/mm^2^. Best matching histological section of prostatectomy specimen (**G**) with tumor foci outlined by the pathologist (dorsolateral edges are missing because of prior frozen section analysis). In this example, both readers assigned a mean PIRADS score of 4 for T2WI and 5 for DWI for both settings.

Selected image sets (T2WI, DWI and ADC) of patients are given in Figs 1, [Fig f2], [Fig f3], 2 and 3 together with the assigned PIRADS scores.

## Discussion

Our preliminary results revealed significantly higher image quality scores with respect to both localization and staging characteristics when an (additional) ERC was used. These results are in line with other findings at 3 T in healthy volunteers^9^ as well as patients with histopathologically confirmed PCa^10^. High-field MR imaging systems are increasingly used in routine clinical settings with the benefit of higher spatial or temporal resolution^15^. The introduction of artifacts, in particular at 3 T, when using an endorectal coil has been observed by Heijmink *et al*. but could not be confirmed by our results. Both readers reported a similar image quality with no substantial artifacts for both coil settings.

In our study, readers were effectively not blinded to the respective imaging coil because the specific choice can be identified by the shape of the prostate gland and rectal wall. This may have introduced a reader bias with lower quality scores for SC images as previously discussed by Heijmink *et al*.^10^.

With respect to the optimization of 3-T diagnostic performance, only two studies have so far directly compared the diagnostic benefit for PCa detection and localization of endorectal MRI with that of a pelvic phased-array coil alone^10^,^16^. In both studies, the diagnostic accuracy with a dedicated ERC was found to be superior. As shown in the study of Heijmink *et al*., the diagnostic accuracy largely depended on the individual experience with endorectal 3-T MRI by showing good sensitivities for advanced (73–88%) and poor results for less experienced readers (13–50%)^10^. The preliminary 3-T results presented here suggest a potential diagnostic benefit of an additional ERC only for the slightly more experienced reader and only with respect to the performance per patient. A possible explanation might be that both readers have so far always read prostate 3-T MRI with additional ERC and never without. Furthermore, all images were obtained with the same sequence parameters which were primarily optimized for a protocol with additional ERC. We deliberately did not adjust our scan parameters for the SC acquisitions in the first part of our MRI examination. Prior studies, however, have shown that acquisition parameters need to be adjusted to the specific setting to guarantee an optimal image quality and diagnostic performance^16^,^17^. This might be an additional explanation for the lower quality scores and somewhat weaker diagnostic performance of the SC images.

Both readers assigned more cancer-suspicious (PIRADS ≥ 4) lesions and also found a higher *number* of true (histopathologically confirmed) tumor lesions with ERC. This finding held independently for both low-risk (GS ≤ 6) and intermediate and high-grade cancers (GS ≥ 7). One reader (B) showed a significant difference in the detection rate of peripheral zone cancers and—most likely because of their higher prevalence—overall as well. It could be argued that ERC imaging at 3 T will generally detect more indolent PCa foci which could potentially lead to overtreatment. On the other hand, there is currently still no non-invasive method to accurately assess tumor aggressiveness. Lesion size, morphological and functional MR information are so far the only established parameters for non-invasive tumor characterization and important parameters for therapy strategies like active surveillance.

The high number of pT2c stages resulted in many low-grade findings (GS = 6) being categorized as high risk such that a stratification against risk was statistically not feasible for our study group. For smaller diameters (≤10 mm), reader A reported more suspicious lesions (both true and false positives) with ERC while the percentage of correctly identified lesions was similar for SC and ERC, respectively. This trend was not seen in the other subgroup (with larger lesions) suggesting a negligible impact of the coils used, albeit only for this particular reader.

DWI is commonly part of multiparametric MRI protocols for the detection of PCa^11^. Over the last years, absolute ADC values have been increasingly considered as a potential measure to distinguish between malignant and benign lesions in the prostate. Different studies, however, have already demonstrated substantial variations of absolute ADC values as a function of imaging equipment and methodology, in particular MRI system and field strength, imaging coils and choice of b-values, which makes it difficult to define predictive ADC thresholds^18^,^19^,^20^,^21^,^22^,^23^,^24^. Our results also suggest that ADC values may depend on the particular receiver coil used for imaging. Normalized cancer ADC values relative to the contralateral side may potentially be more reliable and partially compensate for differences in data acquisition and processing^23^. Such an analysis was beyond the scope of this study and should ideally be validated by different researchers on a larger number of patients.

Our validation study had a number of limitations. This work was generally biased by both readers knowing that all patients had biopsy-proven PCa and were scheduled for prostatectomy at our Urology Department. Like for other studies, our results are not be representative for the general patient population because they were obtained on a limited number of patients at a single institution. Owing to the low prevalence of patients (5 of 41, 12%) with locally advanced disease, the impact of imaging coils on local staging performance could not be analyzed reliably. Reader B correctly detected all patients with extracapsular tumor growth with ERC and gave a higher score (5.0 vs. 3.3) for the quality of identifying minimal capsular penetration when an ERC was used. Furthermore, only patients scheduled for prostatectomy underwent MR examination at our institution. More aggressive cancers are less likely to be confined to the organ and considered for surgical treatment. Despite the resulting underrepresentation of high-grade tumors in our study, typical for prostatectomy patients, the relatively large number of pT2c stages gave rise to an overrepresentation of low-risk tumors here. It should be noted that routine PCa localization often involves dynamic contrast enhancement (DCE) imaging and sometimes MR spectroscopic imaging in addition to T2WI and DWI because these functional techniques have been reported to yield higher diagnostic accuracy^25^,^26^,^27^. Our protocol did not allow for repeated DCE or MR spectroscopic imaging in the same session. In addition, a later examination was not feasible because the majority of patients underwent prostatectomy the day after MRI. The spatial mapping of findings between whole-mount step sections and MR images is generally a non-trivial task and prone to errors because of inherent difficulties in histological sample preparation as well as variability introduced by subjective visual inspections.

In conclusion, the subjectively rated 3-T MR image quality related to the detection, localization and staging of prostate cancer was improved with a dedicated organ coil. In terms of diagnostic performance, the numbers of true cancer foci and of patients with cancer were higher but also that of false positive results.

## Additional Information

**How to cite this article:** Gawlitza, J. *et al*. Impact of the use of an endorectal coil for 3 T prostate MRI on image quality and cancer detection rate. *Sci. Rep.* **7**, 40640; doi: 10.1038/srep40640 (2017).

**Publisher's note:** Springer Nature remains neutral with regard to jurisdictional claims in published maps and institutional affiliations.

## Figures and Tables

**Figure 2 f2:**
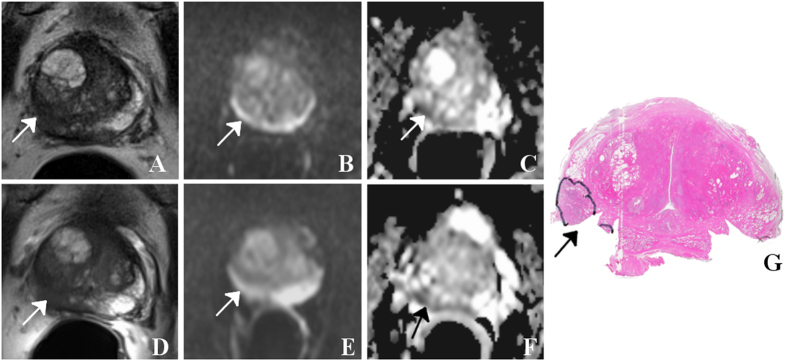
65 year-old patient with prostate-specific antigen level of 56 ng/ml and GS 7 (3 + 4) prostate cancer (arrows) with a diameter larger than 1.5 cm, mainly located in the right peripheral zone (segment 1p/2p): Using surface coils only (**A**) and an additional endorectal coil (**D**), the tumor is visible as a hypointense mass on T2WI. For both settings, features on DWI (**B** and **E**, respectively) are less pronounced while ADC maps (**C** and **F**, respectively) indicate a small corresponding region with restricted diffusion independent of the choice of imaging coils. Best matching histological section of prostatectomy specimen (**G**) with tumor foci outlined by the pathologist (dorsolateral edges are missing because of prior frozen section analysis). In this example, both readers assigned a mean PIRADS score of 4 for T2WI and 4 for DWI for both settings.

**Figure 3 f3:**
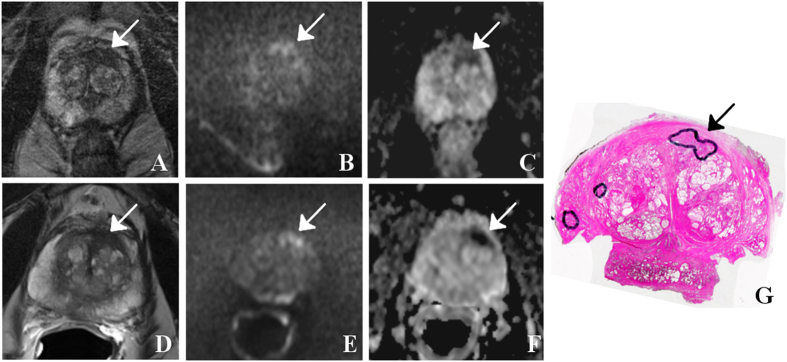
71 year-old patient with prostate-specific antigen level of 8.2 ng/ml and GS 7 (3 + 4) prostate cancer (arrows) in the left transitional and peripheral zone (segment 3a/9a/14as): The tumor is visible as a hypointense signal on T2WI with both surface coils only (**A**) as well as additional endorectal coil (**D**), shows a corresponding focal signal increase on DWI with b-value of 800 sec/mm^2^ (**B** and **E**, respectively) and low values on ADC maps (**C** and **E**, respectively). T2WI suggested extracapsular tumor growth, which was confirmed by histopathology (stage pT3a). Best matching histological section of prostatectomy specimen (**G**) with tumor foci outlined by the pathologist (dorsolateral edges are missing because of prior frozen section analysis).

**Table 1:** Patient characteristics.

Parameter	Value
Number of patients	41
PSA value (median, range) [ng/ml]	11.5 (0.6–56)
Age (average, range) [years]	64 (48–74)
Stage T2a	4
Stage T2b	2
Stage T2c	30
Stage T3a	2
Stage T3b	3
Gleason Score (mean, range)	6.7 (5–8)
Gleason grade 3 + 2	1
Gleason grade 3 + 3	14
Gleason grade 3 + 4	17
Gleason grade 4 + 3	5
Gleason grade 4 + 4	4

PSA, Prostate specific antigen.

TNM, classification of malignant tumors.

**Table 2:** Comparison of mean image quality scores for 3-T MRI in 41 patients using surface coils only (SC) or in combination with an endorectal coil (ERC) for two readers (A and B).

	Reader A		Reader B	
	SC	ERC	SC	ERC
**Related to Localization**				
(a) Discrimination between peripheral and transitional zone	3.3	4.0***	3.9	4.7***
(b) Visibility of the peripheral zone	3.5	4.0**	3.7	4.7***
(c) Visibility of the transitional zone	3.6	4.2***	3.2	4.4***
(d) Visibility of lesion	3.0	3.5*	2.7	4.1***
(e) Visualization of internal architecture of the central gland	3.2	3.9*	3.1	4.3***
**Related to Staging**				
(f) Delineation of prostatic capsule	4.4	4.1**	4.0	4.7***
(g) Visualization of neurovascular bundle	4.4	4.0**	3.9	4.8***
(h) Visualization of rectoprostatic angle	4.4	4.0**	3.9	4.8***
(i) Presence of minimal capsular penetration	1.9	1.9^ns^	2.8	4.4***
**Artifacts**				
(j) Motion artifacts	4.0	3.9^ns^	4.6	4.8^ns^
(k) Other artifacts (aliasing, susceptibility)	4.8	4.7^ns^	4.6	4.8^ns^

^ns^Not significant (p ≥ 0.05).

^*^Significant (p < 0.05).

^**^Significant (p < 0.01).

^***^Significant (p < 0.001).

**Table 3:** Prostate cancer foci and corresponding Gleason Scores detected by both readers when using surface coils alone or in combination with an endorectal coil.

	Reader A		Reader B	
	SC	ERC	SC	ERC
Prostate cancer	38	46	33	56
Mean size (range) [mm]	11.9 (6–29)	9.9 (6–17)	14.8 (8–29)	15.0 (6–27)
GS = 5	1	1	0	1
GS = 6	11	17	9	23
GS = 7	21	23	22	28
GS = 8	5	5	2	4

SC, Surface coils; ERC, Endorectal coil; GS, Gleason Score.

**Table 4:** Detection rates of prostate cancer foci with respect to their localization (transitional versus peripheral zone) by using surface coils alone or in combination with an additional endorectal coil.

	Reader A		Reader B	
	SC	ERC	SC	ERC
Cancer foci	78% (38/49)	74% (46/62)^ns^	69% (33/48)	86% (56/65)*
PZ	80% (33/41)	81% (38/47)^ns^	73% (24/33)	93% (40/43)*
TZ	63% (5/8)	53% (8/15)^ns^	60% (9/15)	73% (16/22)^ns^

TZ, Transitional zone; PZ, Peripheral zone; SC, Surface coils; ERC, Endorectal coil.

^ns^Not significant (p ≥ 0.05).

^*^Significant (p < 0.05).

**Table 5:** Detection rates of prostate cancer foci with respect to their risk category (low, intermediate and high risk^14^) by using surface coils alone or in combination with an additional endorectal coil [**R2**].

	Reader A		Reader B	
	SC	ERC	SC	ERC
Cancer foci	78% (38/49)	74% (46/62)	69% (33/48)	86% (56/65)
Low risk	— (1/2)	— (1/2)	— (1/2)	— (3/4)
Intermediate risk	— (1/2)	— (0/3)	— (0/2)	— (2/4)
High risk	80% (36/45)	79% (45/57)	73% (32/44)	89% (51/57)

SC, Surface coils, ERC, Endorectal coil, —, percentage not given due to very low number of cases.
